# Mechanisms for Combined Hypoxic Conditioning and Divergent Exercise Modes to Regulate Inflammation, Body Composition, Appetite, and Blood Glucose Homeostasis in Overweight and Obese Adults: A Narrative Review

**DOI:** 10.1007/s40279-022-01782-0

**Published:** 2022-11-28

**Authors:** Chris Chow Li Tee, Matthew B. Cooke, Mee Chee Chong, Wee Kian Yeo, Donny M. Camera

**Affiliations:** 1 Division of Research and Innovation, National Sports Institute of Malaysia, Kuala Lumpur, Malaysia; 2 Sport and Exercise Medicine Group, Swinburne University, Room SPW224, Mail H21, PO Box 218, Hawthorn, VIC 3122 Australia

## Abstract

Obesity is a major global health issue and a primary risk factor for metabolic-related disorders. While physical inactivity is one of the main contributors to obesity, it is a modifiable risk factor with exercise training as an established non-pharmacological treatment to prevent the onset of metabolic-related disorders, including obesity. Exposure to hypoxia via normobaric hypoxia (simulated altitude via reduced inspired oxygen fraction), termed hypoxic conditioning, in combination with exercise has been increasingly shown in the last decade to enhance blood glucose regulation and decrease the body mass index, providing a feasible strategy to treat obesity. However, there is no current consensus in the literature regarding the optimal combination of exercise variables such as the mode, duration, and intensity of exercise, as well as the level of hypoxia to maximize fat loss and overall body compositional changes with hypoxic conditioning. In this narrative review, we discuss the effects of such diverse exercise and hypoxic variables on the systematic and myocellular mechanisms, along with physiological responses, implicated in the development of obesity. These include markers of appetite regulation and inflammation, body conformational changes, and blood glucose regulation. As such, we consolidate findings from human studies to provide greater clarity for implementing hypoxic conditioning with exercise as a safe, practical, and effective treatment strategy for obesity.

## Key Points

**Table:** 

Combining exercise and normobaric hypoxia for a short-term period (2–12 weeks) may have synergistic (positive) effects to mediate multiple beneficial health responses in overweight and/or individuals with obesity including reductions in fat mass and improvements in lipid profiles, blood glucose regulation, and insulin sensitivity.
Overweight and/or individuals with obesity may significantly benefit from hypoxic conditioning by achieving a desired cardiometabolic stimulation with lower exercise intensities, and thus a lower mechanical load imposed on the musculoskeletal system, compared with exercise undertaken in normoxia. However, there is no current consensus in the literature regarding the optimal combination of exercise variables, such as the mode, duration, and intensity of exercise, as well as the level of hypoxic conditioning, to maximize health responses with hypoxic conditioning.
Most of the literature investigating combined hypoxic conditioning and exercise in overweight and/or individuals with obesity has incorporated aerobic exercise as the training modality with less focus on combined high-intensity interval training or resistance training with hypoxic conditioning. Therefore, from the available evidence covered in this narrative review, aerobic-based exercise at intensities and duration ranges of between 60 and 70% maximal oxygen uptake and 60–90 min, respectively, combined with moderate levels of hypoxia (approximately 2000–3000 m), appear to provide the most consistent benefits in systemic and skeletal markers for improved appetite, inflammation, and blood glucose regulation, and body composition changes.

## Introduction

Overweight and obesity, defined as a body mass index ≥ 25 and ≥ 30 kg/m^2^, respectively, are associated with impaired metabolic homeostasis, reduced insulin sensitivity [1], postprandial lipid metabolism [2], loss of muscle mass, and increased accumulation of visceral adipose tissue [3], largely attributable to physical inactivity [4]. Regular physical activity/exercise exerts numerous health benefits such as improved cardiovascular fitness, induced anabolic (e.g., increased muscle mass) [5] and metabolic adaptations (e.g., enhanced mitochondrial biogenesis and substrate metabolism) [6–8], and reduced levels of circulating pro-inflammatory markers that reduce all-cause mortality and improve lifespan and quality of life [9, 10].

The American College of Sports Medicine and American Heart Association recommend individuals aged between 18 and 65 years should undertake moderate-intensity continuous training (60–75% maximal heart rate) for a minimum of 30 min 5 days a week or vigorous-intensity training for a minimum of 20 min 3 days a week [11]. However, not all overweight and/or individuals with obesity are able to achieve such physical activity levels required to experience the health benefits from exercise. For instance, it has been reported that overweight and/or obese populations lose their enjoyment of exercise when the exercise intensity is 10% greater than a self-selected speed [12]. Additionally, the excess weight carried by overweight individuals and/or individuals with obesity may increase joint stresses (i.e., hip, knee, ankle) and mechanical demand during simple weight-bearing tasks such as walking [13]. Such increased mechanical constraint (i.e., ground reaction force during walking/running) during weight-bearing activities may also increase the risk of sustaining musculoskeletal injuries and associated pathologies such as lower back pain and osteoarthritis [14–16]. Consequently, there is substantial interest to investigate how overweight and/or obesity populations can attain and maximize the numerous health benefits of exercise by utilizing various therapeutic aids in combination with exercise. Such aids can include nutritional supplementation and pharmaceuticals, as well as exposure to normobaric hypoxia (simulated altitude via reduced inspired oxygen fraction), which has recently emerged as a new therapeutic modality [17].

Hypoxic conditioning is defined as an exposure to hypoxia combined without (passive) and with exercise training (active) to elicit a reduction in oxygen supply to body tissues, which decreases oxygen saturation of the arterial blood [18]. For the purposes of this review, ‘low’ levels of hypoxia are defined as exposure up to 2000 m, ‘moderate’ hypoxia being exposure between 2000 and 3000 m, ‘high’ hypoxia being exposure of between 3000 and 5500 m, and ‘severe’ hypoxia being exposure of above 5500 m [19]. It is well established from epidemiological data that permanent residence in hypobaric hypoxia (terrestrial altitude with a lower barometric pressure) is associated with improvements in blood pressure [20], lower mortality rates, and reduced incidence of obesity prevalence [21–23]. Indeed, a recent meta-analysis showed reductions in fat mass at moderate and extreme altitudes, indicating a lower risk for obesity and a loss of excess weight with prolonged and lifelong exposure to such altitudes [24]. Developing technological devices focused on recreating hypoxic exposure analogous to high-altitude environments through a hypoxic room/tent/chamber by changing either the fraction of oxygen (normobaric hypoxia) or barometric pressure (hypobaric) or wearing a mask (hypoxicator) connected to a gas-mixing device that provides a gas mixture with a reduced oxygen fraction are becoming increasingly popular [25].

Special focus, particularly in the last decade, has been directed towards the intermittent use of such devices and chambers during exercise in the context of obesity [26–28]. Previous studies have reported significant reductions in fat mass and improvements in cardiometabolic health markers including blood lipid profiles [29] and glucose regulation [30], insulin sensitivity [31], and cardiovascular fitness [32] have been reported in healthy individuals performing aerobic-based exercise with hypoxic conditioning (HC). Improvements in glucose tolerance and insulin sensitivity have also been observed in overweight individuals with type 2 diabetes mellitus (T2D) when a single bout of aerobic exercise was performed under moderate hypoxia (i.e., ~ 3000 m) [33, 34].

Evidence also exists demonstrating walking in a hypoxic environment at a slower preferred walking speed (PWS) compared with normoxia-induced similar responses in walking mechanics and energetics in individuals with obesity aged in their mid-30 s, suggesting HC could be effective in increasing the physiological stimulation without a corresponding elevation in stress on locomotion systems [35]. This finding has several important implications. First, overweight and individuals with obesity typically exhibit altered absolute ground reaction forces, joint loads, and forefoot pressures compared with non-obese adults [13]. Additionally, PWS has been consistently reported to be 10–15% slower in obese compared with lean individuals, which would mean overweight and/or individuals with obesity need to walk faster than their PWS in order to increase their exercise intensity and thus augment caloric expenditure for weight loss [36]. Therefore, combined exercise and HC can provide an avenue to reduce muscle/joint load and mechanical strain while concomitantly achieving higher metabolic stress to maximize weight loss and cardiopulmonary adaptation in overweight individuals and/or individuals with obesity [17, 37–39].

Considerable heterogeneity in physiological responses to combined exercise and HC currently exists with numerous studies reporting a similar magnitude of responses in weight and fat mass loss, blood lipid profiles, and insulin sensitivity between exercise performed in hypoxic or normoxic conditions [26, 31, 40, 41]. In addition, intermittent exposure to severe hypoxia has been linked to increased blood pressure [42, 43] and reduced cardiovascular health [44] and may play a chronic pathogenic role in obesity-induced inflammation often seen in obesity [45, 46], although this contention remains equivocal [47–49]. Such observations may relate to the ‘hormesis phenomenon’ where high concentrations/doses of hypoxia may produce detrimental physiological responses compared with lower doses of hypoxia, which may induce beneficial adaptations that provide associated cells and tissues more resilience to the hypoxic stress [50, 51]. In this regard, research study designs utilizing the hormesis concept have incorporated intermittent hypoxia conditioning and intermittent hypoxia-hyperoxia conditioning programs involving several minutes of hypoxic breathing alternated with normoxia or hyperoxia, respectively, for maximizing beneficial health and performance adaptation responses with combined exercise and HC [50]. Such approaches extend upon more traditional altitude training methods including “Live High-Train High” and “Live High-Train Low” and “Live Low-Train High”, which involved longer periods of hypoxic exposure [52, 53].

Recently, studies have begun to elucidate the biological mechanisms underpinning such changes in lipid and glucose metabolism when exercise and HC are combined, specifically in adults with overweight and/or obesity [54]. In this regard, alterations in cell signaling pathways regulating blood glucose regulation along with systemic inflammation and appetite hormones may each be implicated in the beneficial effects associated with exercise and HC in overweight and/or individuals with obesity [55, 56]. Accordingly, the principal aim of this review was to critically evaluate emerging evidence of the key cellular and circulatory markers that are modulated by HC when combined with divergent forms of exercise in overweight and/or adults with obesity. While most studies combining exercise with HC have incorporated aerobic-based programs performed at low-to-moderate intensities, for the first time, we compile growing research literature incorporating high-intensity interval training (HIIT) and resistance exercise with HC and compare the effectiveness of these different modalities for promoting exercise adaptations important for overall health.

We conducted a thorough PubMed search of the literature up until December 2021 that examined the effects of different hypoxic variables and exercise modes using search terms such as ‘passive or active hypoxic conditioning,’ ‘HIIT,’ ‘resistance and/or aerobic-based exercise,’ and ‘overweight and/or obese’. First, studies investigating the responses of appetite and inflammation markers to exercise and HC, including ‘myokines,’ are discussed because of the central role of these processes in the development of obesity. Subsequently, how differences in exercise and HC protocols divergently mediate obesity-induced changes in body composition, appetite hormone regulation, and cell signaling pathways regulating skeletal muscle glucose metabolism are also interrogated. We have specifically focused on these mechanisms considering adipose tissue a major source of inflammatory cytokines with obesity and that such obesity-induced, chronic low-grade tissue inflammation can cause dysregulated glucose metabolism, ultimately culminating in insulin resistance and T2D [57, 58]. Moreover, appetite regulation via ‘peripheral’ mechanisms involving hormones released by the gut and digestive systems is an important regulator of energy balance/imbalance that can contribute to weight regulation [59]. The literature related to studies conducted in overweight and/or individuals with obesity is the primary focus, although studies recruiting healthy or recreationally active individuals are also discussed considering the developing nature of this research field as well as for comparison with overweight individuals and/or obese individuals of measured responses. Readers are referred to several recent excellent reviews that have discussed the combined effects of HC and divergent exercise modes in athletic populations [60] and individuals with T2D [61]. Considering the increasing population incidence of obesity and the need for innovative strategies to combat this deleterious condition, the findings from these studies are of interest to exercise physiologists and sports clinicians by providing a current mechanistic basis for the efficacy of exercise and HC to improve health and well-being outcomes in overweight adults and/or adults with obesity.

## Effects of Exercise Mode and Hypoxic Conditioning on Systemic Inflammation

Overweight and obesity are often associated with chronic low-grade inflammation that has been implicated in the pathogenesis of obesity-related conditions such as insulin resistance, atherosclerosis, and tumor growth [62]. High levels of fatty acids and triglycerides along with ectopic fat storage in sedentary individuals and overweight individuals and/or individuals with obesity lead to an excessive deposit of lipid metabolites (i.e., long-chain fatty acyl-CoA and diacylglycerol) in skeletal muscle [63]. This is accompanied by elevated plasma levels of conventional pro-inflammatory markers such as interleukin (IL)-6, tumor necrosis factor (TNF)-α, and C-reactive protein as well as local inflammation in the skeletal muscle microvasculature of overweight and/or individuals with obesity [64–66]. It is widely accepted that the immune system and the pro/anti-inflammatory cytokine balance can be altered in response to exercise [67]. An inverse relationship between regular physical activity and inflammatory markers including interleukin-6 (IL-6), TNF-α, and adiponectin has been demonstrated in human cross-sectional studies [68–70], revealing the potential anti-inflammatory role of exercise.

More recently, the contraction-induced release of the ‘myokine’ IL-6 is proposed to exert positive effects on skeletal muscle, bone health, and low-grade chronic inflammation via a combination of its role in increasing lipid oxidation and anti-inflammatory effects [71, 72]. Such anti-inflammatory effects of IL-6 are in part mediated via its regulation of IL-1ra and IL-10 [73]. Inhibition of TNF-α by IL-6 and increases in adiponectin levels have been shown to improve glucose uptake by activating the adenosine monophosphate-activated protein kinase signaling pathway [65, 66, 74].

Furthermore, the exercise-induced release of IL-6 is believed to be different between severe illnesses (e.g., T2D and cardiovascular disease) given that anti-inflammatory cytokine expression (e.g., IL-6, IL-1ra, and IL-10) is also elevated with such conditions [69]. In contrast, pro-inflammatory cytokine expression (e.g., TNF-α and IL-1β) does not consistently increase in response to exercise [75, 76] although some studies have reported increased TNF-α expression post-exercise with high-duration stressful activities such as marathon running [77, 78]. In addition, adiponectin has recently been shown to exert anti-inflammatory properties by suppressing both nuclear factor kappa-light-chain-enhancer of activated B cells (NF-κB) inflammatory signaling and reactive oxygen species [79, 80]. With this in mind, elevated levels of circulatory adiponectin may also be indicative of improved health and metabolic outcomes through its multiple protective effects on various cell types and insulin-sensitizing actions [81].

The effects of passive hypoxia on inflammatory responses are equivocal [82, 83]. Hypoxic exposure has been shown to increase levels of circulating pro-inflammatory cytokines expression in individuals with mountain sickness [84]. During three consecutive overnight stays at a high altitude (3458 m) followed immediately by an overnight stay (22 h) at 4559 m, plasma IL-6, IL-1ra, and C-reactive protein levels were increased, implicating a local inflammation response [85]. In contrast, when hypoxia was combined with a single bout of aerobic exercise, no significant differences were observed in circulating levels of TNF-α or IL-1 following 60-min cycling at low-moderate intensity (40–60% of maximal oxygen uptake, VO_2_max) performed in normoxia or moderate hypoxia (2800 m) in overweight [86] individuals [82] (Table 1). At a higher intensity (70% of VO_2_max) and with more severe hypoxia (4500 m), circulatory levels of TNF-α also remained unchanged immediately and 2 h post-treadmill exercise was performed to exhaustion, though IL-6 expression was higher at both timepoints [87]. Hagobian and colleagues [88] found a similar expression profile following a 193-min bout of cycling exercise performed at a moderate exercise intensity (55% of VO_2_max) in severe hypoxic conditions (4300 m), indicating IL-6 may be more sensitive to combined exercise and hypoxic stress compared with TNF-α. Conversely, 60 min of moderate-to-high intensity exercise (70% of VO_2_max) at severe hypoxia (4200 m) increased both pro-inflammatory and anti-inflammatory systemic cytokine expression including IL-6, TNF-α, IL-1ra, and IL-10 immediately post-treadmill running exercise in healthy volunteers [89] (Table 1). These increases in anti-inflammatory cytokines expression (i.e., IL-1ra and IL-10) alongside increased pro-inflammatory cytokines may have occurred as a mechanism to counteract the increase in TNF-α induced by hypoxic stress.

**Table 1:** Effects of passive hypoxia exposure or combined hypoxic condition and resistance, HIIT, or aerobic exercise on systemic pro-inflammatory and anti-inflammatory markers

Study	Participant cohort	Hypoxic conditions	Exercise protocol	Major findings
*Acute exercise or passive (hypoxia)*				
Britto et al. [98]	Male Active, healthy (1) *n* = 10 normoxia (2) *n* = 10 hypoxia	FiO_2_ ~ 14.0%, simulated altitude: ~ 3000 m	Resistance exercise: leg knee extension 8 sets of 8 reps @ 80% of 1-RM	**↑** TNF-⍺ (2)* ↑ IL-6 (2)*
Blegen et al. [86]	Male Overweight, healthy *n* = 9	FiO_2_ ~ 14.6%, simulated altitude: ~ 2800 m	Aerobic exercise: 60-min cycling @ 40–60% VO_2_max	↔ TNF-⍺ ↔ IL-1
Caris et al. [87]	Male Overweight, healthy *n* = 9	FiO_2_ ~ 13.5%, simulated altitude: ~ 4500 m	Aerobic exercise: 3 × 70% of VO_2_peak running until exhaustion	↑ IL-6* ↔ TNF-⍺
Hagobian et al. [88]	Male Recreationally trained, healthy *n* = 18	FiO_2_ ~ 12.1%, simulated altitude: ~ 4300 m	Aerobic exercise: 193-min cycling @ 55% VO_2_max	↑ IL-6* ↑ CRP* ↔ TNF-⍺
Hartmann et al. [85]	Male/female Healthy (1) *n* = 11/1 (2) *n* = 7/3	FiO_2_ (1) ~ 14.4%, (2) ~ 11.8%, simulated altitude: (1) ~ 3458 m, (2) ~ 4559 m	Passive: (1) 3 overnight stays (2) 1 overnight stay	↑ IL-6 (1) and (2)* ↑ IL-1ra (1) and (2)* ↑ CRP (1) and (2)*
Santos et al. [89]	Male Healthy *n* = 9	FiO_2_ ~ 12.5%, simulated altitude: ~ 4200 m	Aerobic exercise: 60-min cycling @ 70% VO_2_max	↑ IL-6* ↑ TNF-⍺* ↑ IL-1ra* ↑ IL-10*
*Prolonged/chronic exercise or passive (hypoxia)*				
Richardson et al. [90]	Male/female *n* = 27/15 (1) *n* = 9/5 control (2) *n* = 9/5 normoxia (3) *n* = 9/5 hypoxia	FiO_2_ ~ 15.0%, simulated altitude: ~ 2500 m	Sprint interval: 6 times/week for 2 weeks @ 30-s ‘all-out’ effort with 4-min recovery	↑ IL-6 (2) and (3)* ↑ TNF-⍺ (2) and (3)* ↑ VO_2_peak (2) and (3), ↔ (1) ↑ TTE (2) and (3), ↔ (1) ↑ AT; W.kg^−1^ (2) and (3), ↔ (1) ↑ AT (3), ↔ (1) and (2)
Żebrowska et al. [91]	Male Healthy *n* = 12 (1) *n* = 6 normoxia (2) *n* = 6 hypoxia	FiO_2_ ~ 15.2%, simulated altitude: ~ 2500 m	HIIT exercise: 3 times/week for 3-weeks @ 6 × 120% of lactate threshold with 5-min recovery	↔ IL-6 (1) and (2) ↔ TNF-⍺ (1) and (2) ↑ VO_2_max (2)*

*1-RM* 1-repetition maximum, *AT* anaerobic threshold, *AT; W.kg*^*−1*^ power at anaerobic threshold, *CRP* C-reactive protein, *FiO*_*2*_ fraction of inspired oxygen, *HIIT* high-intensity interval training, *IL-1* interleukin-1, *IL-1ra* interleukin-1 receptor antagonist, *IL-6* interleukin-6, *IL-10* interleukin-10, *reps* repetitions, *TTE* time to exhaustion, *TNF-⍺* tumor necrosis factor-⍺, *VO*_*2*_*max* maximal oxygen uptake, *VO*_*2*_*peak* peak oxygen uptake, ↑ increased, ↓ decreased, ↔ no change, *significant changes

A study by Richardson and colleagues showed short-term (6 times/week for 2 weeks) sprint interval training (4–7 repetitions for a 30-s maximal effort) at moderate hypoxia (~ 2500 m) increased IL-6 and TNF-α abundance (Table 1) [90]. In contrast, no significant differences were observed in IL-6 and TNF-α following short-term (3 times/week for 3 weeks) HIIT cycling (6 repetitions for 5 min at 120% of the lactate threshold) at moderate hypoxia (~ 2500 m) [91]. Interestingly, this latter study was undertaken in healthy adults compared to the work by Richardson and colleagues [90] in which untrained participants were recruited. It is possible the selective increase in IL-6 and TNF-α expression observed by Richardson et al. [90], as opposed to the work by Żebrowska and co-workers [91], may relate to a pro-inflammatory response induced as a result of the unfamiliarity with the induced contractile stress owing to the exercise-naïve status of the participants, indicating exercise training history/status may be an important regulator of inflammatory responses with combined exercise and hypoxic exposure.

**Fig. 1:**
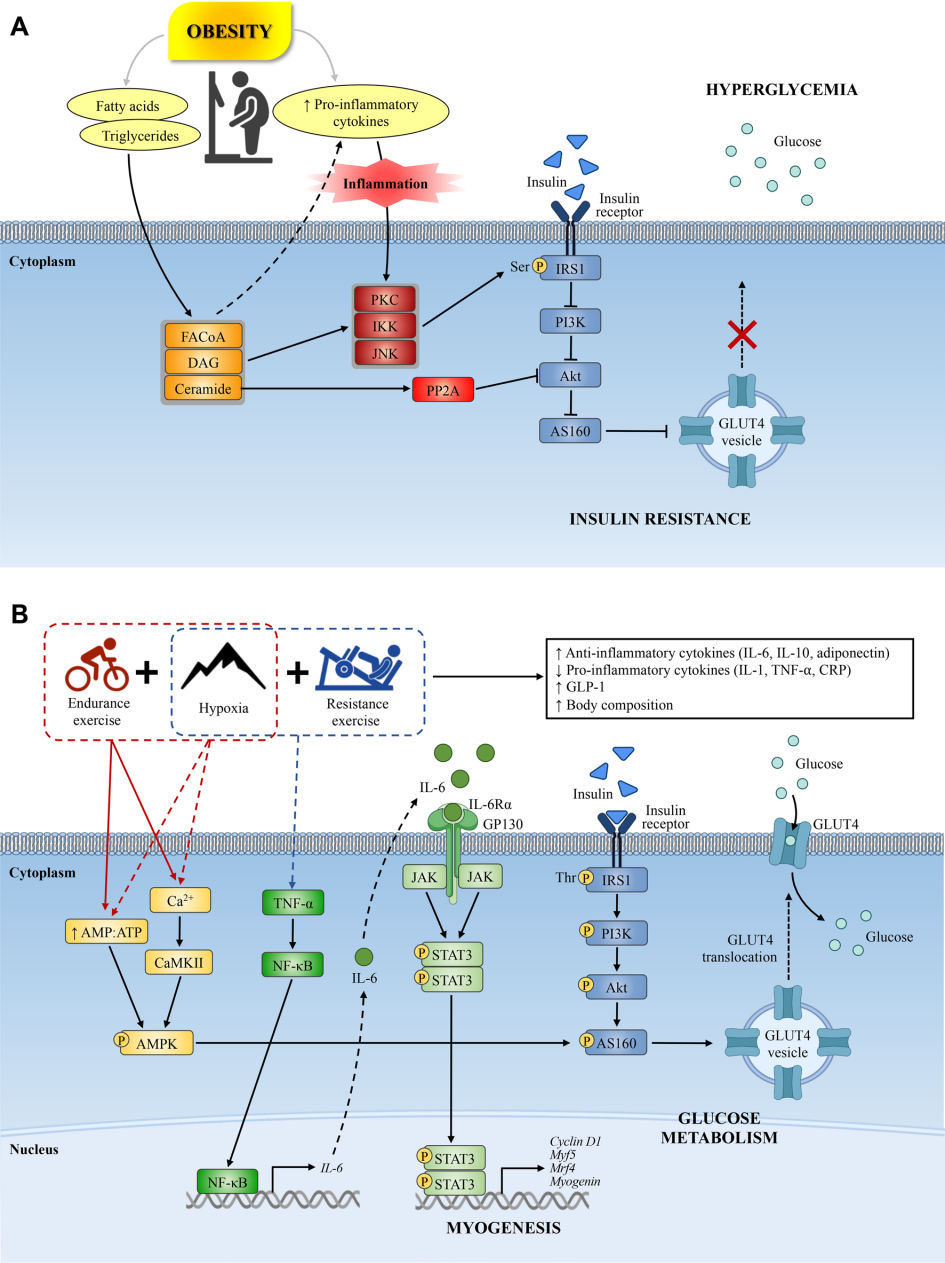
**A** Overweight/obesity-induced insulin resistance: overweight individuals and/or obese individuals with metabolic syndrome present with an excessive accumulation of lipid metabolites and increased plasma levels of pro-inflammatory cytokines. Local inflammation of the microvasculature in skeletal muscle activates the serine kinases protein kinase C, IκB kinase (IKK), and Jun amino-terminal kinase (JNK) that phosphorylate the insulin receptor substrate 1 (IRS1) on serine residues leading to inactivation of IRS1 and downstream inactivation of the insulin signaling cascade. Ceramide accumulation also prevents activation of the insulin signaling cascade in skeletal muscle and reduces insulin activation of AS160 and subsequent glucose transporter-4 (GLUT4) translocation and glucose uptake. **B** Putative mechanisms implicated in the regulation of glucose transport and myogenesis with combined hypoxic conditioning (HC) and exercise training. Exercise stimulates skeletal muscle release of interleukin-6 (IL-6), which further inhibits actions of pro-inflammatory cytokines and increases levels of anti-inflammatory cytokines and glucagon-like peptide-1 (GLP-1). Aerobic-based exercise and hypoxic conditioning have been shown to stimulate GLUT4 translocation, potentially via increases in adenosine monophosphate-activated protein kinase (AMPK)-mediated signaling. Resistance exercise combined with HC may promote myogenesis through regulation of the TNF-ɑ/NF-κB/IL-6/STAT3 pathway. *Akt* protein kinase B, *AMP* adenosine monophosphate, *AS160* Akt substrate of 160 kDa, *ATP* adenosine triphosphate, *Ca2* + calcium, *CaMKll* calcium/ calmodulin-dependent protein kinase II, *CRP* C-reactive protein, *DAG* diacylglycerol, *FACoA* long-chain fatty acyl-CoA, *GP130* glycoprotein 130, *IL-6Ra* interleukin-6 receptor, alpha, *JAK* Janus kinase, *Mrf4* myogenic regulatory factor-4, *Myf5* myogenic factor-5, *NF-κB* nuclear factor kappa-light-chain-enhancer of activated B cells, *PI3K* phosphoinositide 3 kinase, *PKC* protein kinase C, *PP2A* protein phosphatase 2A, *Ser* serine kinase, *STAT3* signal transducer and activator of transcription-3, *Thr* threonine, *TNF-α* tumor necrosis factor-α, ↑ increase/upregulation, ↓ decrease/downregulation; *solid lines* denote established pathways/mechanisms; *broken lines* denote putative pathways/mechanisms

Considerably fewer studies have measured inflammation-mediated responses with hypoxia and resistance exercise compared to those with aerobic-based exercise. Resistance exercise can reduce the risk of low-grade inflammation-related conditions including obesity, insulin resistance, and metabolic disorders [92]. Moreover, negative correlations between circulating levels of IL-6 and TNF-α and muscle mass have been reported, indicating the regulation of these inflammatory markers might be important to maintaining muscle mass and health [37, 93]. Resistance training performed in normoxia between 6 and 12 weeks (2–3 times/week) has been shown to reduce basal levels of TNF-α and IL-6 levels and improve insulin signaling pathway regulation [94]. Such findings may play an important role in glucose metabolism via increased skeletal muscle glucose transporter 4 (GLUT4) protein expression and a decrease in low-grade systemic inflammation [92, 95]. Peake and co-workers [96] reported a selective increase in IL-6 after a single bout of 3 h of submaximal intensity (10% of maximal isometric strength) elbow flexor exercise but not at maximal intensity (100% of maximal isometric strength) in overweight individuals at normoxia. In addition, they observed an increase in TNF-α expression post-exercise (1, 3, and 24 h) at both sub-maximal and maximal intensities. Following 12 months of resistance training (2 times/week, 2–3 sets of 8–12 repetitions at 60–80% of 1-repetition maximum [RM]), increased adiponectin and reduced C-reactive protein expression was observed in overweight individuals at normoxia [97]. To the best of our knowledge, there is only one study that has investigated changes in inflammatory markers after resistance training in hypoxia. A single bout of resistance training (1-legged knee extension consisting of 8 sets of 8 repetitions at 80% of 1-RM with a 2-min rest between sets) performed during moderate hypoxia (~ 3000 m) increased both IL-6 circulatory and messenger RNA (mRNA) levels, as well as TNF-α mRNA levels [98]. While the mechanistic implication of such findings is unclear and requires further investigation, it has been postulated that increases in IL-6 and TNF-α with combined resistance exercise and HC may play a role in muscle hypertrophy via a TNF-α/NF-κB/IL-6/signal transducer and activator of transcription-3-dependent myogenesis pathway. Specifically, TNF-α can induce NF-κB nuclear translocation, which controls IL-6 transcription [99] and subsequent activation of signal transducer and activator of transcription-3 along with its nuclear localization by IL-6 [100]. Signal transducer and activator of transcription-3 subsequently regulates the expression of key proteins involved in skeletal muscle differentiation and proliferation (i.e., cyclin D1, Myf5, MRF4, and myogenin) [100], thus providing a putative pathway for resistance training undertaken in a hypoxic environment to promote myogenesis (Fig. 1). Further mechanistic studies incorporating resistance training-induced contractile activity with different levels of hypoxia are warranted to support this hypothesis.

In summary, combined HC and resistance-based or endurance-based exercise has been shown to induce increases in both pro-inflammatory and anti-inflammatory cytokines. Such heterogeneity in responses may be a result of an acute, transient, and non-specific bodily response to the concurrent stress imposed by exercise and HC, potentially as a mechanism for the cardiopulmonary and vascular systems to adjust to the altered external environment. Moreover, most studies investigating the effects of exercise on inflammatory signaling performed in hypoxic environments have recruited young healthy adults and incorporated aerobic-based exercise training. As such, a greater focus on investigating diverse combinations of exercise variables (mode, duration, and intensity), particularly resistance training, and hypoxic load/duration to optimize anti-inflammatory responses in overweight and/or individuals with obesity of all ages represents an important area of future research.

## Effects of Exercise Mode and Hypoxic Conditioning on Body Composition and Appetite Hormone Regulation

A reduction in body fat is an important goal in weight loss and/or weight management for overweight individuals and/or individuals with obesity because of the positive relationship between increased body fat and chronic inflammation, insulin resistance, and cardiovascular complications [101]. Despite long-term exposure to high-severe hypoxia (> 5000 m) over multiple weeks to months decreasing muscle mass in healthy adults [102], HC alone may be an effective stimulus to induce shifts in fuel utilization and lead to a negative energy balance that could promote weight loss. Indeed, passive acute (3-h) and short-term (1-week) HC at ~ 80% oxygen saturation has been reported to increase energy expenditure and alter fuel utilization by reducing carbohydrate oxidation (− 31% and − 49%, respectively) and increasing fat oxidation (+ 44% and + 29%, respectively) [103] (Table 2). The hypoxia-inducible factor (HIF) is a key transcription factor that regulates several genes that promote metabolic-related adaptations to hypoxia (i.e., glycolytic enzymes and glucose transporter) [104]. Hypoxia-inducible factor-1α, leptin, ghrelin, and glucagon-like peptide-1 (GLP-1) are among several systemic markers implicated in appetite regulation that can be modulated by HC and thus, potentially play a role in mediating beneficial metabolic responses such as glucose homeostasis and insulin sensitivity [105]. Hypoxia-inducible factor-1α is a transcription factor established in the hypoxia signaling pathway through its role in promoting angiogenesis [106, 107]. Thus, HIF-1α is important for enhancing skeletal muscle function by helping match the delivery of oxygen and nutrients with the metabolic needs of myofibers, particularly during exercise [108, 109]. Earlier evidence showed significant increases in hypoxia-sensitive genes HIF-1α (7.8-fold increase at the protein level) and leptin (29.0-fold increase at the mRNA level) in human adipocytes after 4 h of acute hypoxia exposure in vitro [104]. In line with human studies, both passive (an overnight sleep at 2700 m followed by 4 weeks at 4100 m) [110] and active (a single bout of 50 min aerobic running at 70% of VO_2_max and 18 min HIIT running at 90% of VO_2_max at moderate hypoxia [~ 2980 m]) [111] HC stimulate HIF-1α production and have been associated with an increase in leptin and reduction in ghrelin levels [112]. Such responses would lead to appetite suppression, theoretically resulting in a lower energy intake concomitant with increased energy expenditure that occurs at high altitudes, a phenomenon known as ‘altitude anorexia’ [17]. In support of this, findings from a meta-analysis of 28 studies indicate hypoxia exposure decreases hunger sensation compared with normoxia, an effect linked to decreased blood acylated ghrelin levels [113].

**Table 2:** Effects of passive hypoxia exposure or combined hypoxic condition and resistance, HIIT, or aerobic exercise on markers of appetite regulation and body composition

Study	Participant cohort	Hypoxic conditions	Exercise protocol	Major findings
*Acute exercise or passive (hypoxia)*				
Snyder et al. [110]	Healthy *n* = 25	FiO_2_ ~ 12.5%, simulated altitude: ~ 4100 m	Passive: 17-h exposure	↑ Leptin* ↑ GLP-1
Workman and Basset [103]	Male Overweight *n* = 11	80% oxygen saturation	Passive: (1) 3-h exposure (2) 3 h × 1 week	↓ CHO oxidation* ↑ Fat oxidation* ↑ Energy expenditure* ↔ HR (1) and (2) ↔ BP (1) and (2)
Bailey et al. [111]	Male Healthy *n* = 12	FiO_2_ ~ 14.5%, simulated altitude: ~ 2980 m	Aerobic and HIIT exercise: 50 min of running @ 70% of VO_2max_ 6 × 3 min of running at 90% of VO_2_max	↓ Ghrelin* ↔ GLP-1
Morishima and Goto [123]	Male Healthy *n* = 8	FiO_2_ ~ 15.0%, simulated altitude: ~ 2700 m	Passive: 7-h exposure	↓ Postprandial ghrelin* ↔ AUC ghrelin ↔ Leptin ↔ GLP-1
*Prolonged/chronic exercise or passive (hypoxia)*				
Rausch et al. [126]	Male/female Obese *n* = 10/22 (1) *n* = 6/10 control (2) *n* = 4/12 hypoxia	FiO_2_ ~ 15.0%, simulated altitude: ~ 3500 m (exercise) FiO_2_ ~ 12.2%, simulated altitude: ~ 4500 m (rest)	Aerobic exercise: 90-min endurance exercise × 2 times/week × 8 months @ 65–70% of HR_max_ followed by a 90-min resting period	↔ Leptin (1) and (2) ↔ Adiponectin (1) and (2) ↔ BMI (1) and (2) ↔ Peak power output (1) and (2) ↔ HR (1) and (2) ↑ BP (1) and (2)*
Netzer et al. [129]	Male/female Obese *n* = 22/10	FiO_2_ ~ 15.0%, simulated altitude: ~ 2500 m	Aerobic exercise: 90-min endurance exercise × 3 times/week × 8 weeks @ 60% of HR_max_	↓ Body mass* ↓ TG ↓ LDL
Nishimura et al. [148]	Male Untrained: (1) *n* = 7 normoxia (2) *n* = 7 hypoxia	FiO_2_ 16.0%, simulated altitude: ~ 2100 m	Resistance exercise: 2 × 6-weeks @ 70% of 1-RM	↑ Arm muscle mass (2)* ↑ Arm muscle strength (2)*
Wiesner et al. [32]	Male/female (1) *n* = 8/13 normoxia (2) *n* = 10/14 hypoxia	FiO_2_ 15.0%, simulated altitude: ~ 2740 m	Aerobic exercise: 3 times/week × 4 weeks running @ 65% of VO_2_max	↓ Lactate at AT (2)* ↓ Fasting insulin and HOMA-IR index (1) and (2)* ↓ Body fat (2)* ↔ TG (1) and (2) ↔ HDL (1) and (2) ↔ LDL (1) and (2) ↑ VO_2_max (1) and (2)
Camacho-Cardenosa et al. [131]	Female Overweight *n* = 82 (i) Aerobic interval (normoxia) *n* = 13 (ii) Aerobic interval (hypoxia) = 15 (iii) Supramaximal interval (normoxia) *n* = 15 (ii) Supramaximal interval (hypoxia) *n* = 15	FiO_2_ ~ 17.2%, simulated altitude: ~ 2500 m	Aerobic exercise: 3 times/week for 12 weeks (i) Aerobic interval: 3 min @ 90% W_max_ followed by 3-min recovery @ 55–65% of W_max_ (ii) HIIT: 30 s @ 130% of W_max_ followed by 3-min recovery at 5–65% of W_max_	↓ body fat in HIIT group* ↑ muscle mass in HIIT group*
Klug et al. [127]	Male Obese *n* = 23 (1) *n* = 11 normoxia (2) *n* = 12 hypoxia	FiO_2_ ~ 15.0%, simulated altitude: ~ 2500 m	Aerobic exercise: 60 min: (3 × 15-min intervals walk with 5-min break for recovery) for 6 weeks @ 50–60% of HR_max_	↑ Postprandial energy expenditure (2)* ↓ Blood pressure (1) and (2)* ↓ Waist circumference (1) and (2)* ↓ LDL (1) and (2)* ↔ TG ↓ HR (1) and (2)
Yang et al. [125]	Male/female Obese, healthy *n* = 22/18	FiO_2_ ~ 14.7%, simulated altitude: ~ 2700 m	Passive: sleep in the chamber for 10-h daily for 4 weeks	↓ BMI* ↑ Lean body mass* ↑ GLP-1* ↔ Ghrelin
Kong et al. [130]	Female Overweight, sedentary *n* = 24 (1) *n* = 13 normoxia (1) *n* = 11 hypoxia	FiO_2_ ~ 15.0%, simulated altitude: ~ 2500 m	HIIT exercise: 4 times/week for 5 weeks @ 8 s × 60 reps at maximal effort with 12-s recovery	↑ TC/HDL (1) and (2)* ↑ TG/HDL (1) and (2)* ↔ Body composition (1) and (2) ↔ Leptin (1) and (2) ↑ VO_2_peak (1) and (2)
Debevec et al. [29]	Male Healthy *n* = 14 (1) *n* = 6 sedentary (2) *n* = 8 exercise	FiO_2_ ~ 13.9%, simulated altitude: ~ 4000 m	Aerobic exercise: 60-min cycling × 2 times × 10 days @ 50% peak power output	↔ GLP-1 (1) and (2) ↔ Leptin (1) and (2) ↔ Ghrelin (1) and (2)
Morishima et al. [128]	Male Overweight, sedentary (1) *n* = 11 normoxia (2) *n* = 9 hypoxia	FiO_2_ ~ 15.0%, simulated altitude: ~ 2800 m	Aerobic exercise: 60-min cycling × 3 times/week × 4 weeks @ 55% of VO_2_max	↔ Energy intake ↓ Leptin in both group* ↔ GLP-1 ↔ Ghrelin ↔ VO_2_max (1) and (2) ↓ Workload (2)* ↔ HR (1) and (2)
Park et al. [134]	Male Obese, elderly *n* = 24 (1) *n* = 12 normoxia (2) *n* = 12 hypoxia	FiO_2_ ~ 14.5%, simulated altitude: ~ 3000 m	Aerobic and resistance exercise: 90–120 min of combined running, cycling and resistance × 3 times/week × 12 weeks 60 min of aerobic exercise @ 60–70% of HR_max_ 10–15 reps of whole-body resistance exercise @ 6–7 OMNI-Resistance Exercise Scale of Perceived Exertion	↑ Body composition (body mass, body fat and fat-free mass) (1) and (2)* ↑ Physical fitness (1) and (2)* ↑ Pulmonary function (1) and (2)*
Torpel et al. [150]	Male/female *n* = 42 Young (1) *n* = 15/2 normoxia (2) *n* = 17/3 hypoxia Older (3) *n* = 9/8 normoxia (4) *n* = 9/10 hypoxia	~ 80–85% oxygen saturation	Resistance exercise: 4 times/week × 5 weeks 3 sets of 15 reps @ (25–40% of 1-RM) 7 CR-10 scale for whole body machine-based resistance exercise	↔ Body composition (1–4) ↑ VO_2_peak (4), ↔ VO_2_peak (1–3)

*AT* anaerobic threshold, *BMI* body mass index, *BP* blood pressure, *CHO* carbohydrate, *FiO*_*2*_ fraction of inspired oxygen, *GLP-1* glucagon-like peptide-1, *HDL* high-density lipoprotein, *HIIT* high-intensity interval training, *HR* heart rate, *HR*_*max*_ maximal heart rate, *LDL* low-density lipoprotein, *reps* repetitions, *TG* triglycerides, *VO*_*2*_*max* maximal oxygen uptake, *VO*_*2*_*peak* peak oxygen uptake, *W*_*max*_ maximal watt, ↑ increased, ↓ decreased, ↔ no change, * significant changes

Leptin acts on neurons in the brainstem and hypothalamus to modulate satiety and the control of reward and aversion [114]. Leptin is a central marker of long-term energy stores for the central nervous system with an established function in regulating the balance between food intake and energy expenditure [115]. Increased plasma levels of leptin have been reported following short-term hypoxia in middle-aged male individuals with obesity, indicating leptin production may be stimulated by hypoxia at high altitudes despite an observed reduction in the body weight of participants [20]. The ‘hunger hormone’ ghrelin also acts on the hypothalamus with roles in appetite stimulation and increased food intake and fat storage [116]. Research over the last two decades has implicated ghrelin in modulating the appetitive response to food cues and feeding regulation [117]. In contrast to leptin, unchanged [20, 118] and decreased [119] levels of ghrelin have been reported at high-altitude/hypoxic environments. Glucagon-like peptide-1 can promote satiety, suppress energy intake, and subsequently prevent weight gain in both human and mouse models [120, 121]. Glucagon-like peptide-1 secretion is typically stimulated by nutrients that have been digested and reach the intestinal L cells [122]. Acute [123] and chronic [124] exercise training in normoxic conditions have also been shown to increase GLP-1 levels. Regarding hypoxic stimuli, passive moderate HC (~ 2700 m), 7 days/week for 4 weeks, has been shown to significantly reduce the body mass index and increase GLP-1 expression in obese adolescents [125] (Table 2). The authors of this work postulated that the observed increase in GLP-1 may be partially mediated by IL-6, a regulator that directly responds to hypoxia via NF-κB activation to positively regulate appetite [125]. Similarly, 17 h of passive severe HC (~ 4100 m) has been shown to increase postprandial GLP-1 levels in young and overweight female individuals, but this did not achieve statistical significance [110]. In contrast, postprandial GLP-1 levels were unchanged following 7 h of passive HC at moderate hypoxia (~ 2700 m) in healthy individuals [123] (Table 2). Collectively, these findings suggest either severe or longer exposure to moderate (passive) HC is required to increase GLP-1 secretion.

Regarding the combined effects of aerobic exercise and HC, GLP-1 levels remained unchanged, while ghrelin levels decreased following a single session of moderate-intensity running (70% of VO_2_max) and HIIT running (6 × 3 min at 90% of VO_2_max) at moderate hypoxia (~ 2980 m) in healthy individuals [111]. Similarly, circulatory GLP-1 levels were unchanged following 10 days of moderate-intensity exercise (two daily 60-min cycle training sessions performed at 50% peak power output) and severe HC (~ 4000 m) in healthy individuals [29]. Subcutaneous adipose tissue mRNA expressions of leptin and adiponectin were also unchanged following long-term (32 weeks) moderate-intensity exercise (cycling, running, or cross-training for 2 times/week at 65–70% maximal heart rate) at severe hypoxia (3500 m) in female and male individuals with obesity [126]. In contrast, several weeks of moderate HC (i.e., 2500–2800 m) combined with moderate-intensity cycling (55–60 VO_2_max/60% maximal heart rate) or HIIT (‘all-out’ effort) exercise (3–4 times/week over 4–8 weeks) induced several beneficial effects in overweight and/or individuals with obesity including increased postprandial energy expenditure [127] and GLP-1 expression [128], as well as reductions in triglycerides and low-density lipoproteins [129]. However, these findings are not universal with others observing no changes in plasma ghrelin, energy intake [128], and body composition [130] with short-term (4–5 weeks) moderate-intensity exercise or HIIT performed at moderate hypoxia (~ 2500 m) (Table 2). In this regard, combined aerobic-based exercise and HC have been shown to induce greater reductions in body weight and improvements in pulmonary function and the basal metabolic rate compared with normoxia in individuals with obesity [129, 131–134]. Specifically, one study in sedentary and overweight younger adults reported a two-fold magnitude increase in VO_2_peak following 5 weeks of HIIT performed in hypoxia compared with normoxia [130]. Similarly, another study in middle-aged adults with obesity showed that 8 weeks of combined HC and low-intensity endurance exercise induced a significantly greater weight loss (− 1.14 kg) compared with a normoxic (− 0.03 kg) environment [129]. These findings were supported by a recent systematic review and meta-analysis in overweight individuals and/or individuals with obesity demonstrating a significant reduction in fat mass when aerobic/HIIT-based exercise was performed under hypoxia but not in normoxia [135]. This same systematic review and meta-analysis also demonstrated a trend (*p* = 0.08) for higher muscle mass gain with combined HC and exercise (predominantly aerobic-based cycling and treadmill running/walking) compared to normoxia, indicating overall greater beneficial effects on body composition when exercise is performed under hypoxia compared with normoxia [135]. However, the additional positive effects of HC in this systematic review and meta-analysis did not extend to waist circumference, blood lipid levels, and waist/hip ratio, with a similar magnitude of responses observed between exercise conducted in hypoxia or normoxia [135]. Similarly, other studies have observed no additional benefits of hypoxia on exercise training-induced muscle mass [127, 136] and weight loss in adolescent [137, 138] or middle-aged [32, 35, 40, 130, 139, 140] individuals with obesity. Specifically, body weight and body mass index decreased to a similar magnitude following 4 weeks of aerobic exercise undertaken at 65% VO_2_max performed in either hypoxia (oxygen fraction = 15%) or normoxia, despite a greater reduction in body fat content and increase in fat-free mass in the hypoxia group [32]. Over longer intervention periods, Gatterer and colleagues also reported no further weight reduction following 8 months of low-to-moderate intensity aerobic exercise (65–70% heart rate peak) between hypoxia and normoxia conditions in middle-aged adults with obesity [40]. The low-to-moderate exercise intensities may explain the lack of weight change reported in these studies, particularly as it is postulated that higher/maximal intensities may be more beneficial for weight loss [141]. In this regard, exercise modality may also be an important consideration for promoting weight loss with HC. Indeed, long-term HIIT (3 times/week, 12 weeks) undertaken in moderate hypoxia (~ 2500 m) has been shown to reduce body fat and increase muscle mass in female individuals with overweight and/or obesity [131]. The post-intervention reduction in fat (and total body) mass observed in this work was well beyond the 3% change in body weight considered clinically significant [36], providing strong support for HIIT under normobaric intermittent hypoxia to reduce body fat content and increase muscle mass in overweight women and/or women with obesity. While the implication of such findings beyond the 3-month period of this study remains an area of future investigation, it should be noted that weight loss with conventional exercise/dietary interventions typically plateaus after 6 months [142].

New insight regarding muscle strength and hypertrophy responses with resistance training performed in different levels of hypoxia is starting to form with a postulation that the combination of lower aerodynamic resistance and/or increased anaerobic metabolism enhances motor unit recruitment patterns and metabolic cost at higher altitudes [143]. In this regard, the combination of moderate hypoxia and resistance exercise has been demonstrated to improve aspects of anabolic (muscular strength and hypertrophy) [144–149] and overall body composition [134] (Table 2). These findings agree with other studies incorporating upper and lower body resistance training (70% of 1-RM, 5 × 10 repetitions) between 5 and 8 weeks that have reported a 1–2% reduction in fat mass in severe hypoxia (~ 3000–3900 m), although no statistical significance was noted when compared to resistance training in normoxia [146, 149]. In contrast, 5 weeks of whole-body resistance training (25–40% of 1-RM, 3 × 15 repetitions) in moderate hypoxia (~ 80–85% oxygen saturation) did not improve body composition in overweight young individuals and old individuals [150]. No studies have investigated the combined effects of resistance training and HC on markers of appetite regulation and body compositional changes greater than 10 weeks. However, one recent study investigated combined whole-body resistance (60–70% of 1-RM, 3 × 10–15 repetitions) and aerobic exercise (running and cycling for a total of 60 min at 60–70% of maximal heart rate) in high hypoxia (3000 m) for 12 weeks and showed improved body composition by reducing body weight and body fat, and increasing fat-free mass, in older individuals with obesity [134]. While these findings provide some evidence for resistance training in hypoxia to induce positive body composition changes as evident by decreases in body fat and increases in lean body mass, further interrogation of the cellular mechanisms mediating these responses is still required. In particular, potential changes in muscle protein synthesis and mitochondrial biogenesis with combined resistance exercise and HC are largely unknown and would intuitively be implicated in the beneficial effects on body composition observed currently in the literature. Additionally, whether increases in muscle cross-sectional and strength can be further promoted and potentially optimized with hypobaric (terrestrial altitude) versus normobaric hypoxia (simulated altitude) is another area of future research. Nonetheless, based on current findings, combined HC and resistance training may be a useful strategy to help promote lean mass accrual, which is of particular importance considering the increasing incidence of deleterious effects of sarcopenic obesity with advancing age [151].

In summary, combined exercise and HC does not appear to provide any further beneficial effects on hormonal regulators of appetite, with limited positive effects observed regardless of exercise mode or intensity. Notwithstanding, appetite control is regulated by a complex interplay of neural, endocrine, and nutrient signals/sensors that work concomitantly to mediate both short-term and long-term caloric intake and energy reserves [18]. Thus, further interrogation of such mechanisms is required to better understand how exercise and HC can alter appetite regulation in individuals with obesity. While several studies report greater magnitude responses in weight loss when exercise is undertaken in hypoxia compared with normoxia, these responses are inconsistent across the literature. Such disparity may relate to differences in the intensity, frequency, and volume (i.e., exercise session duration and number of sessions per week) of exercise training combined with HC. Studies targeting higher intensity exercise protocols combined with dietary management are logical steps to better determining the efficacy for HC to induce greater weight loss with hypoxia compared with normoxia. Additionally, current knowledge regarding the efficacy of combining resistance exercise with HC for promoting additional muscle hypertrophy, power, and strength adaptation is still in its infancy, although current findings are promising regarding the promotion of anabolic adaptive responses. This represents an important area of future investigation, particularly as obesity in older adulthood occurs in conjunction with sarcopenia (sarcopenic obesity) and is associated with impaired skeletal functionality [152].

## Effects of Exercise Mode and Hypoxic Conditioning on Blood Glucose Regulation

Skeletal muscle is the major site of insulin-stimulated glucose uptake in the postprandial state, taking up 70–90% of the glucose from the blood [153, 154]. This uptake is mainly facilitated by either the solute carrier family 2, which consists of the facilitative glucose transporters or the solute carrier family 5, which comprises select sodium-dependent glucose co-transporters. In individuals with obesity, high levels of circulating inflammatory markers and local inflammation of the microvasculature in skeletal muscle result in the inactivation of the insulin signaling pathway that can decrease the translocation of GLUT4 to the plasma membrane for glucose uptake [155] (Fig. 1).

Endurance-based exercise is well established to decrease glycated hemoglobin [156] and fasting blood glucose levels [157, 158]. Mechanistically, aerobic exercise undertaken in normoxia significantly upregulates GLUT4 protein levels and cell signaling pathways regulating its expression [159–163]. Regarding the effects of acute hypoxia in isolation, one study in 11 sedentary overweight male individuals reported a concomitant decrease and increase in glucose and fat metabolism, respectively, following 3-h normobaric hypoxic exposure [103]. In contrast, no significant changes in blood glucose, insulin, and lipid profiles were observed in sedentary individuals, overweight individuals and/or individuals with obesity between passive hypoxic (~ 5700 m; three 1-h sessions per week for 8 weeks) and normoxic exposure [139]. Short-term (1 week, whole day) passive moderate hypoxia exposure (2650 m) reduced glycated hemoglobin levels in individuals with obesity [20]. The possible explanation for the discrepancy in these findings may be related to the metabolic health status of the participants recruited in these studies: the study by Chacaroun and colleagues [139] recruited participants with normal blood glucose levels whereas individuals with T2D were included in the study conducted by Lippl and co-workers [20], suggesting that metabolically compromised individuals may be more sensitive to beneficial effects of passive hypoxic exposure.

When considering the combined effects of HC and aerobic-based exercise, overweight individuals with T2D who performed 60 min of cycling at 90% of the lactate threshold under moderate hypoxia (~ 3000 m) improved insulin sensitivity during a 4-h intravenous glucose tolerance test [33] (Table 3). In a follow-up study, 60 min of cycling at 90% of the individual lactate threshold in moderate hypoxia (~ 3000 m) reduced fasting glucose and insulin in overweight individuals with T2D, while these effects were not seen following 20 min of cycling at 90% of the lactate threshold in moderate hypoxia [164]. Notably, because no control group was included in this study, it is unclear whether these beneficial effects were caused by exercise and the added hypoxia stimulus or purely by the exercise bout. Similarly, 60 min of combined endurance (cycling at 50–90% of maximal aerobic power) and resistance training (50–70% of 1-RM, 4 × 6–15 repetitions) 3 times/week for 6 weeks with moderate hypoxia (~ 2800 m) reduced plasma insulin levels and improved glucose tolerance in adolescents with obesity, although there was no statistical significance between the hypoxic and normoxic conditions [138]. Running on a treadmill at 65% of VO_2_max 3 times/week for 4 weeks under moderate hypoxia (~ 2740 m) improved fasting insulin levels compared with baseline in overweight individuals and individuals with obesity [32]. This finding contrasts with other work showing neither passive moderate hypoxic (~ 2700 m) exposure [41] nor exercise in moderate hypoxia (~ 2740 m) [12 × 1-h cycling at 65% of VO_2_max over 2–4 weeks] improved fasting blood glucose and insulin levels [165]. Taken together, the beneficial effects of combined HC and aerobic-based exercise on blood glucose regulation are equivocal with no consistent findings among studies that have incorporated either similar exercise or hypoxic protocols. Notably, there is a paucity of studies incorporating longer aerobic-based exercise interventions (i.e., > 16 weeks) with HC. Such studies may yield novel strategies for improving blood glucose regulation in individuals with T2D.

**Table 3:** Effects of passive hypoxia exposure or combined hypoxic condition and resistance, high-intensity interval training, or aerobic exercise on blood glucose, insulin and lipid profiles

Study	Participant cohort	Hypoxic conditions	Exercise protocol		Major findings
*Acute exercise or passive (hypoxia)*					
Mackenzie et al. [33]	Male T2D, overweight: *n* = 8	FiO_2_ 14.6%, simulated altitude: ~ 3100 m	Aerobic exercise: 60-min cycling @ 90% LT *Determined in a submaximal cycling test in normoxia (1) 60-min rest in normoxia (2) 60-min rest in hypoxia (3) 60-min cycling in normoxia (4) 60-min cycling in hypoxia		↔ Blood lactate between (1) and (2), ↑ during exercise but ↔ between (3) and (4) ↓ Blood glucose during (2), (3) and greater in (4)* ↑ Insulin sensitivity in (2) compared with (1) and higher in (4) compared with (3). It was higher in (4)*
Mackenzie et al. [164]	Male T2D, overweight: *n* = 8	FiO_2_ 14.7%, simulated altitude: ~ 3100 m (1) 60 min in hypoxia (2) 40 min in hypoxia (3) 20 min in hypoxia	Aerobic exercise: (1) 60-min cycling @ 90% lactate threshold (2) 40-min cycling @ 70 W (128% @ 90% lactate threshold) (3) 20-min cycling @ 140 W (257% @ 90% lactate threshold)		↓ Blood glucose and plasma insulin (1) and (2) 24 h and 48 h later. It was a greater reduction in (1)*
Morishima et al. [41]	Male Sedentary, overweight: *N* = 8 (1) Normoxia (2) Hypoxia	FiO_2_ 15.0%, simulated altitude: ~ 2700 m	Aerobic exercise: 30-min cycling @ 60% of VO_2_max at 8:00, 10:30, and 13:00 (7.5 h exposure)		↔ AUC blood glucose and insulin (1) and (2) ↑ % CHO oxidation (2)* ↑ HR (1) and (2)*
*Prolonged/chronic exercise or passive (hypoxia)*					
Chacaroun et al. [139]	Male/female Overweight/obese: *n* = 24/11 (1) *n* = 7/4 control (2) *n* = 8/5 sustained hypoxia (3) *n* = 10/2 intermittent hypoxia	FiO_2_ 10.3%, simulated altitude: ~ 5700 m		Passive: 60 min × 3 times/week × 8 weeks (1) 60-min ambient air (2) 5-min baseline normoxia, 55-min hypoxia, 5-min recovery normoxia (3) 5-min baseline normoxia, 7 × 5-min hypoxia followed by 3-min normoxia, 7-min recovery normoxia	↔ Blood glucose (1–3) ↔ Insulin (1–3) ↔ TG (1–3) ↔ Total cholesterol (1–3) ↔ HDL (1–3) ↔ LDL (1–3) ↑ Pulmonary function (2) and (3)* ↔ HR (1–3) ↔ BP (1–3)
De Groote et al. [138]	Male/female Obese: *n* = 6/8 (1) *n* = 3/4 normoxia (2) *n* = 3/4 hypoxia	FiO_2_ 15.0%, simulated altitude: ~ 2800 m		Aerobic and resistance exercise: 50–60 min × 3 times/week × 6 weeks Session 1: 12-min cycling (2 min at 50% MAP and 10 min at 70% MAP) Session 2: 2 min at 50% MAP and 5 reps of 1 min at 70% and 1 min at 50% MAP Session 3: 40% MAP with an increase of 10% MAP every 2 min and resistance training (15 reps @ 50% 1-RM + 4 sets of 6 reps @ 70% 1-RM; rest 2 min)	↓ AUC insulin (2)* ↓ Glucose (2)* ↓ TG (2)* ↑ MAP (2)* ↑ Work capacity (2)*
Lippl et al. [20]	Male Obese: *n* = 20	FiO_2_ 15.0%, simulated altitude: ~ 2650 m		Passive: 24-h exposure × 1 week	↓ Fasting blood glucose ↑ Insulin sensitivity
Morishima et al. [165]	Male Sedentary: *n* = 21 (1) *n* = 11, 2 weeks (2) *n* = 10, 4 weeks	FiO_2_ 15.0%, simulated altitude: ~ 2740 m		Aerobic exercise: (1) 60-min cycling for 6 times/week × 2 weeks @ 65% VO_2_max (2) 60-min cycling for 3 times/week × 4 weeks @ 65% VO_2_max	↔ Postprandial glucose in (1) and (2) ↓ AUC postprandial insulin in (2)* ↑ VO_2_max (1)* and (2) ↓ BP (1) and (2)*

*1-RM* 1-repetition maximum, *AUC* area under the curve, *BP* blood pressure, *CHO* carbohydrate, *FiO*_*2*_ fraction of inspired oxygen, *HDL* high-density lipoprotein, *HR* heart rate, *LDL* low-density lipoprotein, *LT* lactate threshold, *MAP* maximal aerobic power, *reps* repetitions, *TG* triglycerides, *VO*_*2*_*max* maximal oxygen uptake, ↑ increased, ↓ decreased, ↔ no change, *significant changes

Resistance exercise training performed in normoxic conditions has also been shown to improve systemic glucose control as evidenced by reduced glycated hemoglobin levels and fasted blood glucose levels following 16 weeks of progressive resistance training (3 times/week, 60–80% of 1-RM, 3–6 sets × 10–15 repetitions) [166, 167]. Additionally, increased sodium-dependent glucose co-transporter 3 mRNA and protein levels have been reported following 16 weeks of resistance training (3 times/week at 60–65% of 1-RM) [168], indicating additional glucose transporter(s) aside from GLUT4 play a role in skeletal muscle uptake with resistance training-induced contraction. Croymans and colleagues [169] reported improved insulin sensitivity, lean body mass, relative strength as well as GLUT4 expression in overweight individuals and/or individuals with obesity after 12 weeks of resistance training (3 times/week) at normoxia. Resistance training for 6 weeks (70% of 1-RM, 4 × 10 repetitions) aided by moderate hypoxia (~ 2100 m) elicited a greater muscle hypertrophy response (cross-sectional area) as compared with normoxia [148]. Such findings may be of potential clinical significance considering glucose tolerance and insulin sensitivity are positively correlated with muscle mass [170]. More recently, De Groote and co-workers [138] found that both moderate hypoxic (~ 2800 m) and normoxic groups induced similar increases in lean muscle mass after combined endurance (cycling at 50–90% of maximal aerobic power) and resistance training (50–70% of 1-RM, 4 × 6–15 repetitions) 3 times/week for 6 weeks, despite the hypoxic group exhibiting a greater glucose tolerance and lower insulin response to an acute glucose challenge. As such, the synergistic effects of hypoxia and resistance training on glucose metabolism may be independent of skeletal muscle hypertrophy induced by resistance training. In this regard, a recent meta-analysis of nine studies in 158 mostly trained participants showed resistance training performed in hypoxia significantly increases muscle cross-sectional area and strength [171]. However, this increase was not additive to increases in muscle cross-sectional area induced in normoxia, which the authors concluded may be owing to differences in resistance training protocols implemented between studies [171]. Thus, the use of more standardized training protocols is needed in order to better elucidate the cellular and systemic mechanisms that may mediate enhanced skeletal muscle glucose uptake with combined HC and resistance training.

## Conclusions and Future Directions

Health, exercise, and nutrition-based associations continually promote the beneficial health effects of exercise for overweight and/or individuals with obesity. Nonetheless, global rates of obesity continue to rise, prompting researchers to devise new and innovative methods for such cohorts in an attempt to increase exercise attendance and adherence to combat the deleterious effects of obesity. In the last decade, increasing numbers of studies have shown that aerobic, HIIT, and even resistance exercise performed under varying levels of hypoxia can augment exercise-associated health and metabolic benefits, including the effects on blood glucose homeostasis, pro-inflammatory and anti-inflammatory responses, markers of appetite regulation, and body composition. Considering the increasing global prevalence of metabolic-related disorders, such findings are meaningful to overweight individuals and/or individuals with obesity who may significantly benefit from exercising in hypoxia by achieving the desired cardiometabolic stimulation with lower exercise intensities and thus lower mechanical load imposed on the musculoskeletal system compared with exercise undertaken in normoxia. To this end, the combination of intermittent hypoxic exposure and exercise provides a potential and translatable avenue for personalizing exercise prescription to these populations to ultimately improve exercise training adherence. However, as also identified in this narrative review, combined exercise and HC have been associated with several pathological effects. Thus, the use of hypoxia during exercise should be administered with care and participants closely monitored for any adverse effects (i.e., acute mountain sickness symptoms) under such conditions. Moreover, considerable variability in physiological responses to combined exercise and HC also exists with multiple studies demonstrating no further health benefits for overweight individuals and/or individuals with obesity exercising in a hypoxic compared to a normoxic environment. In this regard, there is no current consensus in the literature as to which level (i.e., low: 500–2000 m, moderate: > 2000–3000 m, high: 3000–5500 m, or severe: > 5500 m) of HC is optimal when combined with exercise to exert the most beneficial effects on blood glucose regulation and body composition changes without promoting pro-inflammatory responses. This is perhaps unsurprising considering the disparity in exercise protocols and hypoxic loading incorporated between studies. Thus, optimizing hypoxic dose–response, training intensity, training mode, and dietary control is a logical step to maximizing the multiple health benefits of combined exercise and hypoxic conditioning.

**Fig. 2:**
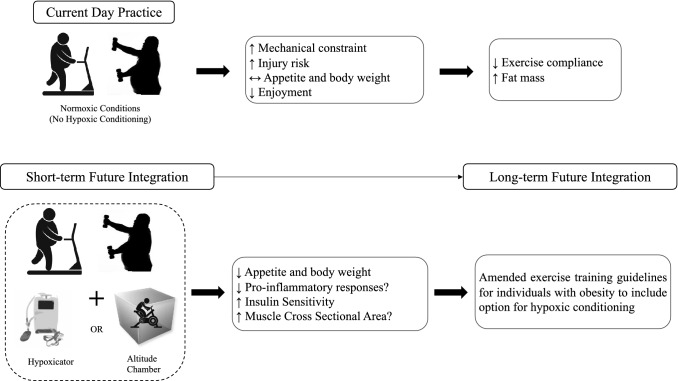
Current and future integration of combined hypoxic conditioning and exercise training for overweight individuals and/or obese individuals with metabolic syndrome. Overweight individuals and/or individuals with obesity have been previously shown to reduce their physical activity levels owing to reduced enjoyment and also because of greater mechanical constraints due to greater joint/tendon stress, thus increasing the risk of injury. Accumulating evidence supports the integration of both resistance and aerobic-based exercise (including high-intensity interval training) and hypoxia as a therapeutic strategy to improve multiple health outcomes, including fat mass loss and enhanced blood glucose regulation, and reduce the mechanical load imposed on the musculoskeletal system compared with exercise undertaken in normoxia. With continued standardization of exercise training and hypoxic variables in future studies, the inclusion of combined hypoxic conditions and exercise performance with hypoxicators or in simulated altitude chambers provides a feasible strategy to assist in combatting the deleterious effects of obesity

Most literature investigating combined HC and exercise in overweight and/or adults with obesity has incorporated aerobic exercise as the training modality with less focus on combined HIIT or resistance training with HC. Therefore, from the available evidence covered in this narrative review, aerobic-based exercise at intensities and duration ranges of between 60 and 70% VO_2_max and 60–90 min, respectively, combined with moderate levels of hypoxia (approximately 2000–3000 m), appear to provide the most consistent benefits for improved appetite, inflammation and blood glucose regulation, and body composition changes. Combined HC and exercise performance within these parameters provide a starting point for health professionals and clinicians to consider for treatment options to promote beneficial health outcomes in overweight individuals and/or individuals with obesity. However, to provide further support for these summations, future studies standardizing the combination of exercise (e.g., intensity, duration, type of exercise) and hypoxic variables (normobaric, hypobaric, dose, and duration) are urgently required. Specifically, fewer studies have incorporated hypoxia and resistance exercise compared with aerobic-based exercise, particularly over several months of training. As many of the anabolic-related adaptations to resistance training are generally evident after 8–12 weeks, it would be interesting to investigate the combined effects of resistance training and HC on health markers, hormone regulation, and physical performance over such durations [172, 173]. In addition to increasing understanding of the different variables that can enhance the beneficial effects of exercise and HC, further studies examining the systemic and myocellular mechanisms mediating such responses in overweight and/or individuals with obesity are required. Elucidating these signaling pathways is crucial to improving the health and well-being of these populations. Another area for future investigation is the incorporation of controlled nutritional and psychological/cognitive-based interventions with HC and exercise programs to maximise improvements in body composition and associated health variables (i.e., blood glucose, lipids). In particular, determining the efficacy for HC to increase exercise adherence in overweight and/or individuals with obesity represents an important area for study, considering regular participation in exercise has been shown to require motivation and enjoyment to maintain adherence in this population cohort [174]. Finally, with technological advances, HC devices such as hypoxicators for home use along with increased accessibility to hypoxic chambers offer significant potential for real-world application as a therapeutic, cost-effective, and accessible treatment strategy for helping overweight individuals and/or individuals with obesity improve health outcomes (Fig. 2).
